# Weeding Out the Culprit: Cannabinoid-Associated Stevens-Johnson Syndrome

**DOI:** 10.7759/cureus.39454

**Published:** 2023-05-24

**Authors:** Jessie Li, Michael Miller, Suha Abu Khalaf, Taylor B Nelson

**Affiliations:** 1 Medicine, University of Missouri, Columbia, USA; 2 Infectious Diseases, University of Missouri, Columbia, USA

**Keywords:** clinical case report, cutaneous adverse drug reaction, cannabis use, toxic epidermal necrolysis (ten), stevens-johnson syndrome (sjs)

## Abstract

We describe a case of Stevens-Johnson syndrome (SJS) in a 32-year-old female who initially presented with a several-day history of worsening rash. Diagnosis of cannabinoid-associated SJS was established following skin biopsy and detailed history-taking of medication and other recreational drug usages. The patient was treated with pain management, antihistamines, and topical steroids with no complications following discharge. There currently exists limited literature describing SJS due to recreational drug usage.

## Introduction

Stevens-Johnson syndrome (SJS) is a hypersensitivity reaction that most commonly manifests as a severe skin reaction caused by medication usage that can occur alongside systemic symptoms [1]. This type of reaction is often triggered by medication usage but can also be secondary to an infection, though in many cases no clear etiology can be identified [2,3]. SJS usually presents with a prodrome of nonspecific systemic symptoms such as fever, chills, myalgia, and photophobia one to three days prior to skin manifestations. The rash itself consists of erythematous macules and patches that progress into bullae that eventually slough. Mortality of this spectrum of disease is high, and treatment options are limited and supportive in nature following discontinuation of the causative agent [2,4].

## Case presentation

A 32-year-old female with a three-day history of a vesicular and maculopapular rash presented with worsening skin lesions despite treatments from an outside hospital. Her medical history included anxiety, irritable bowel syndrome, and degenerative disc disease. Her home medications included buspirone, venlafaxine, and propranolol, all of which she had been taking for over a year with no dosage changes. The rash began as small erythematous and circular eruptions across her chest and neck. These lesions progressed into edematous blisters that later ruptured. The patient had tried over-the-counter diphenhydramine and hydrocortisone, but these medications did not alleviate the pain or itch associated with the rash. The rash grew and gradually became more painful. She sought care at an outside hospital where she was given an intramuscular steroid, diphenhydramine, and famotidine and was then discharged home with a topical steroid and a six-day course of oral steroids. The following morning, the rash progressed to involve her face, arms, chest, abdomen, thighs, and perineum. She returned to the outside hospital and was prescribed doxycycline for suspected tick-borne illness and discharged home. Despite these treatments, the rash continued to progress and involved her eyelids, ears, and perioral region, sparing the mucosal surfaces, palms, and soles of the feet. Due to these worsening symptoms, she presented to our facility. The patient endorsed fever, chills, headache, and mild photophobia. She denied any new prescriptions or recent medication changes and had been taking her psychiatric medications for over a year. She had no new environmental exposures, recent sick contacts, or recent tick bites that could be recalled. She had known allergic rash eruptions with use of penicillin-based antibiotics but had no recent use of antibiotics.

**Figure 1:**
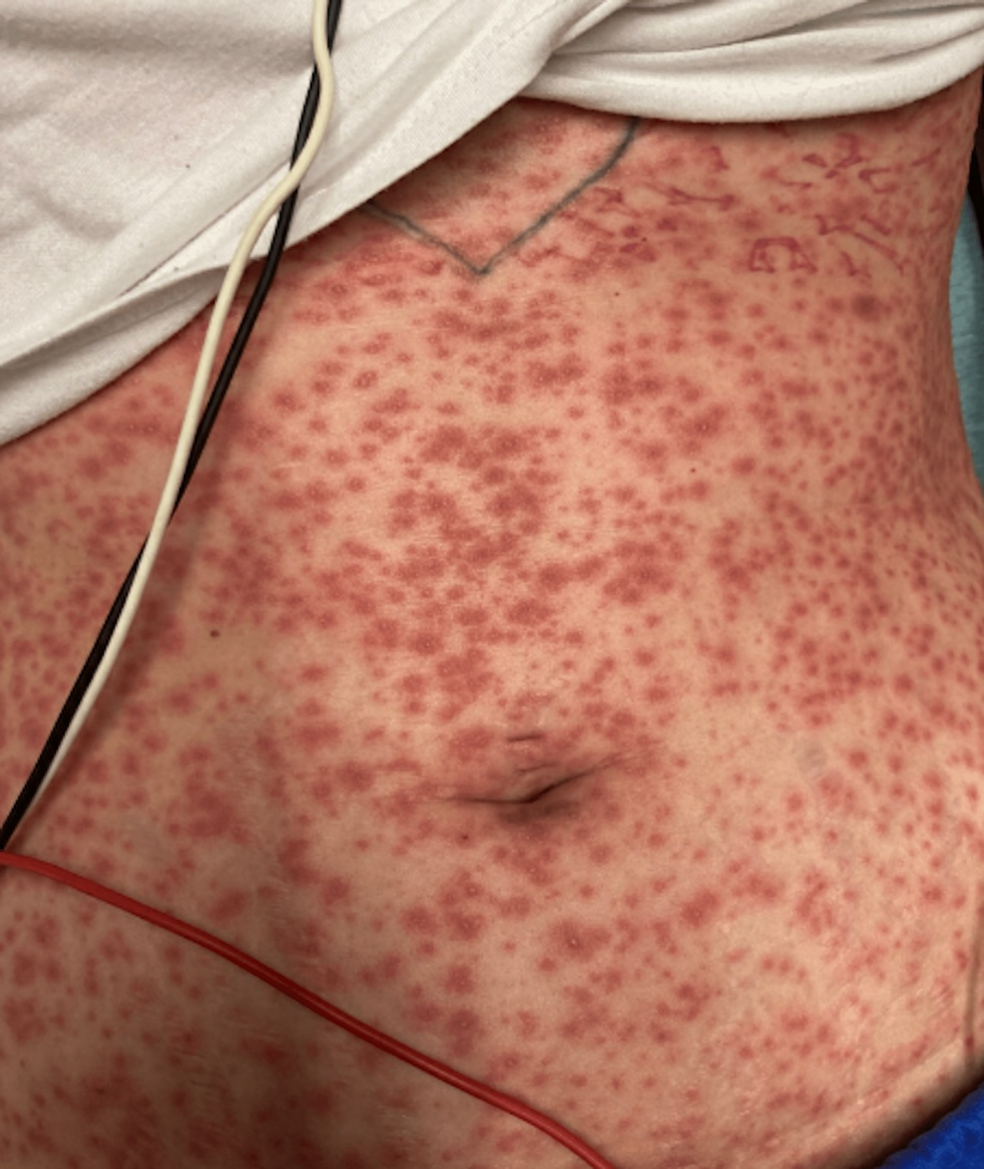
Patient’s Abdomen, Day 3 of Exanthem

**Figure 2:**
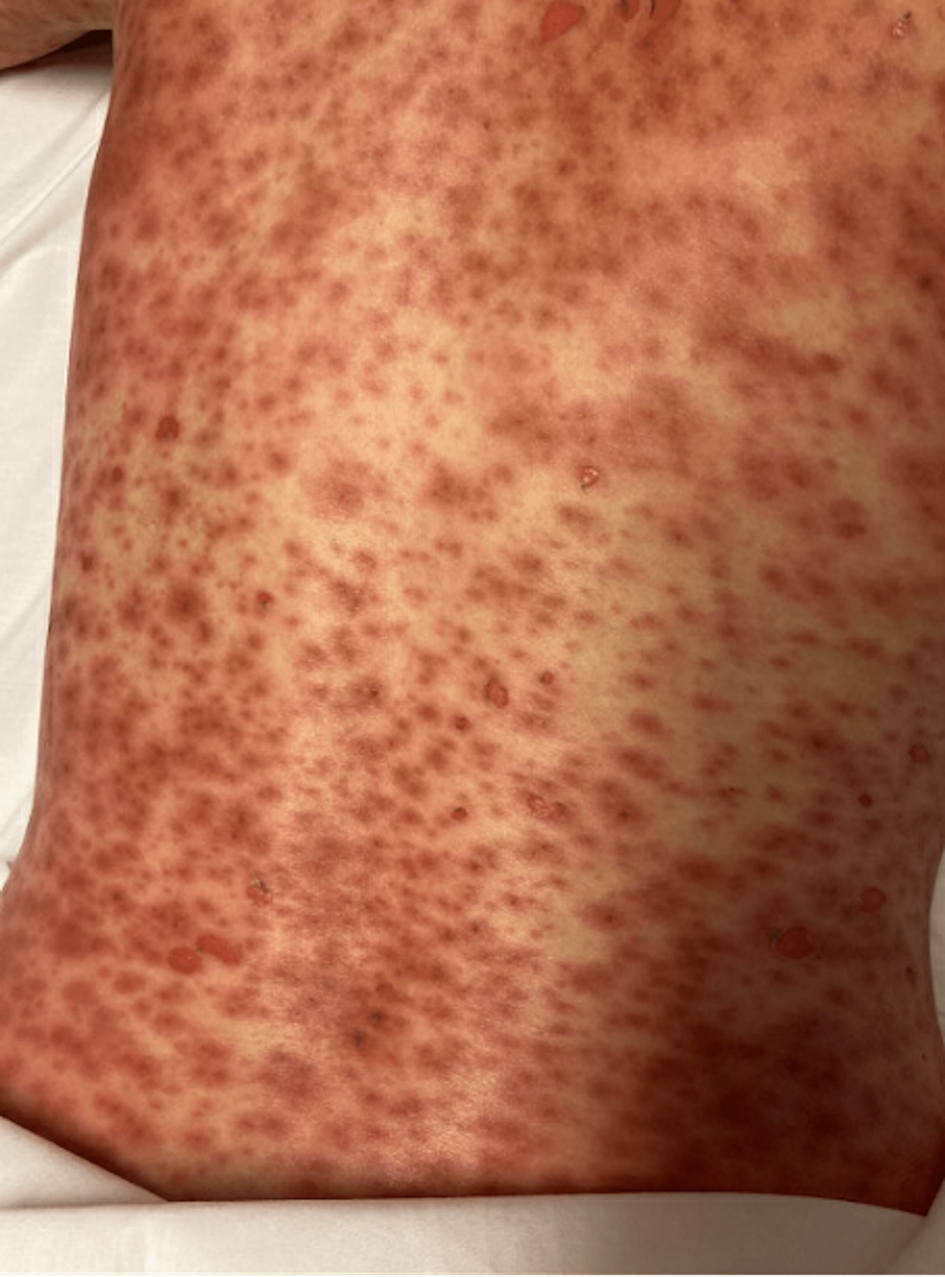
Patient’s Back, Day 5 of Exanthem

On presentation, she was febrile with a maximum temperature of 38.6°C, heart rate of 90 bpm, respiration rate of 15 breaths/minute, and blood pressure of 146/66 mmHg. Left postauricular and left neck lymphadenopathy were appreciated on physical exam. The rash itself consisted of dark, edematous papules that coalesced into plaques. They appeared targetoid and some lesions had a duskier center. Many of these lesions were vesicular, and they seemed to be in different stages (Figure 1, 2). Two punch biopsies were taken from the left abdomen and left upper extremity in the emergency department (ED). Initial laboratory workup of a complete blood count (CBC), complete metabolic panel (CMP), and urinalysis during the ED visit did not reveal any abnormalities. The only significant laboratory value was an elevated C-reactive peptide (Table 1). The patient was admitted to the hospital, given IV pain medication, and started on acyclovir with concern for possible herpesvirus infection. 

**Table 1:** Hospital Lab Values WBC: white blood cells; RBC: red blood cells; Hgb: hemoglobin; Hct: hematocrit; MCV: mean corpuscular volume; RDW SD: red cell distribution width, standard deviation; PLT: platelets; MPV: mean platelet volume; Abs: absolute; ALP: alkaline phosphatase; AST: aspartate transaminase; ALT: alanine transaminase; ESR: erythrocyte sedimentation rate; CRP: C-reactive protein

	Hospital Day 1	Hospital Day 3	Reference Ranges
WBC (x 10^9^/L)	7.22	2.48	3.50-10.50
RBC (x 10^12^/L)	4.54	4.29	3.90-5.03
Hgb (g/dL)	14.3	13.1	12.0-15.5
Hct (%)	41.10	37.30	34.9-44.5
MCV (fL)	90.5	86.9	81.6-98.3
RDW SD (fL)	39.6	38.7	36.4-46.3
PLT (x 10^9^/L)	194	149	150-450
MPV (fL)	9.8	9.7	8.0-12.0
Abs Granulocytes (x 10^9^/L)	6.36	-	1.70-7.00
Abs Immature Granulocytes (x 10^9^/L)	0.02	-	0.00-0.03
Abs Lymphocytes (x 10^9^/L)	0.65	-	0.90-2.90
Abs Monocytes (x 10^9^/L)	0.18	-	0.30-0.90
Abs Eosinophils (x 10^9^/L)	0.00	-	0.05-0.50
Abs Basophils (x 10^9^/L)	0.01	-	0.00-0.30
Abs Neutrophils Manual (x 10^9^/L)	-	1.79	1.70-7.00
Abs Bands Manual (x 10^9^/L)	-	0.02	0.00-0.88
Abs Lymphocytes (x 10^9^/L)	-	0.52	0.90-2.90
Abs Reactive Lymphs Manual	-	0.1	NA
Abs Monocytes Manual (x 10^9^/L)	-	0.05	0.30-0.90
ALP (units/L)	51	148	35-104
AST (units/L)	22	155	<=32
ALT (units/L)	22	255	10-35
ESR (mm/Hr)	6.0	-	0.0-20.0
CRP (mg/dL)	2.11	-	0.00-0.50

Given the nature of this rash, an infectious etiology was considered. The initial infectious differential for this rash was broad and included atypical presentations of eczema herpeticum or erythema multiforme secondary to *Mycoplasma pneumoniae* or herpes simplex virus (HSV). Workup for infection included nucleic acid testing for chlamydia/gonorrhea, HIV 1,2 antigen and antibody screening, syphilis antibody screening, respiratory pathogen panel, various tick-borne pathogens including species of *Ehrlichia, Anaplasma, Borrelia, *and* Rickettsia*, and blood cultures. From the vesicular fluid, polymerase chain reaction (PCR) for HSV, varicella-zoster virus, and *Mycoplasma pneumoniae *was performed as well as a Tzank smear. These tests were all negative. 

On hospital day 2, the rash continued to spread to below the patient’s knees. The patient was afebrile with a temperature of 36.4°C, a heart rate of 85 bpm, and a blood pressure of 133/65 mmHg. Dermatopathology results from lesions on her abdomen and left upper arm identified epidermal necrosis with mononuclear infiltrate, vacuolar interface changes at the dermal-epidermal junction, and single necrotic keratinocytes within the follicular epithelium. Based on these histopathologic findings, the leading differentials from dermatopathology included erythema multiforme, paraneoplastic pemphigus, and SJS secondary to medication or drug use. No evidence of changes secondary to herpetic infection was identified. Due to these findings, the patient’s buspirone and venlafaxine were initially held for concern of worsening her condition. For the patient’s pain and itch related to the rash, scheduled hydromorphone and hydroxyzine were administered. 

On hospital day 3, the patient continued to endorse significant pain from her rash. The rash approached mucosal surfaces but continued to show no mucosal involvement. The patient’s laboratory values were significant for leukopenia, thrombocytopenia, and transaminitis (Table 1), thus raising concern for atypical presentation for tick-borne illness. Following a negative viral workup, acyclovir was discontinued and doxycycline was started. Additionally, the patient reported withdrawal symptoms following the discontinuation of venlafaxine and buspirone. The psychiatric service was consulted and felt it was unlikely that these medications would have precipitated the patient’s reaction as she had been taking them for over a year without recent changes. Thus, the patient was restarted on venlafaxine and buspirone. Dermatology was consulted concerning the patient's rash and recommended 0.05% clobetasol ointment to cover the entire body, and continuous wound care precautions were put in place. Per ophthalmology recommendation, a daily regimen of periocular lubrication and preservative-free artificial tears was initiated. Further discussion with the patient on hospital day 3 revealed that she had used a new strain of cannabis the day before the onset of the rash. This timeline of events favored a necrolytic drug-related eruption secondary to cannabis use.

The patient was closely monitored throughout the rest of her seven-day stay. She was eventually taken off doxycycline due to lesser concern for tick-borne disease. The clobetasol ointment was switched to triamcinolone topical ointment on hospital day 4, which the patient found to be more helpful. She continued receiving acetaminophen and hydromorphone for pain control and hydroxyzine for itching related to her rash. Due to concerns for prolonged fluid loss from vesicle rupture, she additionally was started on continuous intravenous fluids. Over the course of the rest of her stay, the patient’s rash and pain control continued to show improvement. Prior to discharge, the patient was educated on proper wound treatment and avoidance of triggers including vapes and cannabis use. Through phone conversation two days following discharge, the patient reported significant improvement in the rash, new skin growth, and no signs of infection.

## Discussion

SJS and toxic epidermal necrolysis (TEN) are severe cutaneous eruptions that are characterized by disruption of the epidermal-dermal junction leading to severe skin sloughing [1]. The etiology of these syndromes is most frequently related to the use of medications including sulfa derivatives, nonsteroidal anti-inflammatory medications, penicillin-based antibiotics, tetracyclines, cephalosporins, antiepileptics, barbiturates, and recreational drugs [2]. The risk is greatest one to three weeks following new medication exposure, but this can extend for up to eight weeks [5]. Thus in patients who have been taking prescribed medications for a long period of time, such as in this patient’s case, clinical suspicion should be low for these agents being causative and another source should be investigated. Reportedly up to half of all cases of SJS or TEN are idiopathic. The mortality rate overall for the two syndromes is high, estimated to be between 10-50% [6].

A prodromal syndrome of flu-like symptoms, fever, chills, photophobia, conjunctival irritation, and dysphagia often precedes SJS cutaneous eruptions by one to three days [2,7]. The epidermal reaction presents as diffuse erythematous or dusky violaceous patches, targetoid lesions with central necrosis, and scattered bullae and blisters that may progress into erosions or ulcers. The presence of a positive Nikolsky sign is characteristic of this diagnosis. SJS and TEN reactions are typically associated with mucosal involvement, but the absence of mucosal involvement does not preclude the diagnosis [4]. Commonly associated laboratory abnormalities include anemia and leukopenia, particularly neutropenia. Electrolyte abnormalities may present due to excessive sloughing and fluid loss. Elevation of aminotransferases two to three times above the normal limit can be observed in approximately half of TEN cases [8].

This patient’s symptom course aligned with clinical descriptions of SJS/TEN reactions. She experienced prodromal symptoms of fever, chills, and photophobia prior to the onset of skin eruptions. Biopsy findings of these lesions showed different stages of development, healing, and rupture, and cytotoxic findings and vacuolar changes at the dermal-epidermal junction were consistent with histopathologic findings of SJS. While laboratory studies on admission were largely unremarkable, this patient developed leukopenia and elevated transaminase levels a few days after symptom onset. Her infectious workup was ultimately negative, which further supported this diagnosis. The major limitation of SJS/TEN diagnoses was the absence of mucosal involvement. 

Management for SJS/TEN is largely supportive in nature. Immediate steps in management include discontinuation of any suspected triggers, wound care, and pain control. Multidisciplinary consultations including dermatology and ophthalmology are also important aspects of care. There exists conflicting literature and recommendations surrounding other adjunctive therapies, including systemic corticosteroids, intravenous immunoglobulin (IVIG), tumor necrosis factor-alpha (TNF-α) inhibitors, and cyclosporine, for the treatment of SJS/TEN [1,4].

Identification of the trigger of SJS/TEN disease is rarely definitive in nature and requires clinical judgment and detailed history-taking. Given the lack of changes with this patient’s prescription medications and negative infectious disease workup, recreational utilization of a new strain of cannabis in the days preceding symptom onset was determined to be the most likely source of her reaction. Our patient’s specific strain of marijuana was not made available for further analysis due to loss of follow-up. Thus, we were unable to perform more specified testing to determine product composition and possible contaminants. Literature for SJS/TEN related to cannabis use is scarce; thus, this case report represents relatively novel findings. To our knowledge, one other case report of SJS related to cannabinoid product consumption exists [6]. Other published case series and reports document similar cannabinoid skin rashes, but the disease course in these cases was less severe in nature. The reported time course for the onset of these rashes ranged from six hours to nine days following cannabis usage [9-11]. Further research is necessary to better establish the relationship between cannabinoid products and cutaneous reactions including SJS/TEN.

The current landscape of cannabis availability in the United States is largely unregulated, leading to an increased risk of contaminants, substitutions, and undisclosed adulterants. This lack of clarity on the exact composition of cannabis products lends itself to a higher potential for morbidity and mortality with use, including risk for skin reactions such as SJS [12]. Chemical analysis was not conducted on the cannabis product in this case, but generally, the pathogenesis of SJS/TEN reactions is incompletely understood and thought to be similar to the mechanism of type IV hypersensitivity reactions [4]. Movements toward legalization and decriminalization of marijuana in the United States coincide with increased availability of FDA-regulated cannabis products. While regulation cannot eliminate inherent risks of medication and drug utilization, it has been shown to lend itself to safer and more effective use. Thus, having greater oversight within this industry will allow patients and providers to make more informed decisions about cannabinoid product use [12].

## Conclusions

Here, we have described a novel case of SJS associated with cannabinoid usage. This case demonstrates that a detailed history is necessary to delineate exposures in the setting of SJS. Recreational drug usage is unregulated by the FDA and thus poses an unknown risk of hypersensitivity reactions. Further research will provide an increased understanding of the association between cannabis products and SJS. Rapid identification of causative agents with SJS is critical for discontinuation and avoidance to improve clinical outcomes.
